# Neuroprotection with glatiramer acetate: evidence from the PreCISe trial

**DOI:** 10.1007/s00415-013-6903-5

**Published:** 2013-04-16

**Authors:** Douglas L. Arnold, Sridar Narayanan, Samson Antel

**Affiliations:** NeuroRx Research, 3605 University Street, Montreal, QC H3A 2B3 Canada

**Keywords:** Multiple sclerosis, Axons, Magnetic resonance imaging, Magnetic resonance spectroscopy, Clinical trials, N-acetylaspartate

## Abstract

The phase III, multicenter, randomized, placebo-controlled PreCISe trial assessed glatiramer acetate (GA) effects in patients with clinically isolated syndromes (CIS) suggestive of multiple sclerosis (MS). To assess the neuroprotective effect of GA in a subset of patients in the PreCISe trial, we used proton magnetic resonance spectroscopy (MRS) to measure N-acetylaspartate (NAA), a marker of neuronal integrity, in a large central volume of brain. Thirty-four CIS patients randomized to GA 20 mg/day (*n* = 19) SC or placebo (*n* = 15) were included. Patients who relapsed (developed clinically definite MS [CDMS]) were removed from the substudy. NAA/creatine (NAA/Cr) ratios were compared between GA-treated and placebo-treated patients. Twenty patients with CIS had not converted to CDMS and were still in the double-blind phase of the trial at 12 months of follow-up. Paired changes in NAA/Cr differed significantly in patients treated with GA (+0.14, *n* = 11) compared with patients receiving placebo (−0.33, *n* = 9, *p* = 0.03) at 12 months, consistent with a neuroprotective effect of GA in vivo. Patients with CIS who received GA showed improvement in brain neuroaxonal integrity, as indicated by increased NAA/Cr, relative to comparable patients treated with placebo, who showed a decline in NAA/Cr consistent with findings from natural history studies.

## Introduction

The importance of neuroaxonal degeneration as the substrate of chronic irreversible disability in multiple sclerosis (MS) is being increasingly appreciated [1]. The neurological impairment that occurs with MS relapses results primarily from acute conduction block associated with demyelination and inflammation [2]. Resolution of inflammation, along with remyelination of axons that are not transected, and insertion of new sodium channels to allow conduction across segments of axons that remain demyelinated, can lead to reversal of the disability attributable to these factors. However, axons are often transected in acute lesions, and axons that are not transected but remain demyelinated may degenerate due to increased influx of sodium and calcium, loss of trophic support by myelin, and impaired axonal transport and mitochondrial function. Irreversible injury and loss of axons lead to an irreversible loss of nervous system function and, to the extent that cerebral plasticity and redundancy cannot compensate, to irreversible functional impairment and disability. Chronic diffuse inflammation within the CNS [3] probably accelerates the loss of axons.

The integrity of axons in MS can be assessed in vivo based on changes in the signal intensity of the neuronal marker compound, N-acetylaspartate (NAA) [4]. NAA is synthesized by neuronal mitochondria and is localized almost exclusively within neurons, including dendrites and axons. NAA density in the brain can vary due to a change in the relative partial volume of neurons, such as occurs with neuroaxonal loss, or to a change in the concentration of NAA within neurons, such as occurs with sublethal neuroaxonal injury. NAA metabolism is linked to mitochondrial metabolism, and decreases in NAA may be at least partially reversible [5]. This is central to the usefulness of NAA as a marker of neuroaxonal integrity, since mitochondrial dysfunction is often an early accompaniment of injury.

In untreated patients with relapsing-remitting multiple sclerosis (RRMS), the ratio of NAA to creatine concentration (NAA/Cr) determined by magnetic resonance spectroscopy (MRS) is lower than normal [4], and decreases by approximately 5 % per year [6, 7]. This decrease in NAA/Cr, which is dominated by changes in normal-appearing tissue, correlates with increases in EDSS scores more strongly than conventional MRI metrics based on focal white matter lesions [8]. Preliminary data from small studies without randomization or placebo controls have suggested that treatment with glatiramer acetate (GA) may be able to slow or reverse expected declines in NAA/Cr [9–11].

In order to more definitively establish whether GA could slow or reverse the expected decline in NAA over time in patients with a clinically isolated syndrome (CIS), we performed a prospective substudy utilizing MRS in a subgroup of patients participating in a large phase III, multicenter, randomized, placebo-controlled trial of GA in patients with CIS (the PreCISe study [12, 13]).

## Methods

### PreCISe trial

Details of the design and results of the PreCISe trial have been presented elsewhere [12] (The trial is registered with clinicaltrials.gov, number NCT00666224). The protocol and patient consent documents were approved by the institutional review boards and ethics committees of the participating centers. In brief, the trial consisted of a three-year, randomized, placebo-controlled, double-blind comparison of GA 20 mg SC QD and matching placebo in patients with CIS suggestive of MS. A total of 481 patients were randomized at 80 centers around the world. The main inclusion criteria were a single, unifocal clinical attack and a brain MRI showing two or more lesions at least 6 mm in diameter. The primary aim of the study was to assess the efficacy of GA in CIS. Following conversion to clinically definite MS (CDMS) or after 3 years, whichever came first, patients could switch to active treatment with GA for a total treatment duration of 5 years.

The placebo-controlled phase of the trial [12] was terminated early, as a preplanned interim analysis of the results favored treatment with GA over placebo, and the data monitoring committee recommended, with sponsor approval, to offer all patients the opportunity to roll over into the open-label phase of active GA therapy for 2 years.

### MRS substudy

This prospectively planned MRS substudy included 34 patients enrolled at 10 clinical sites in seven countries. All patients gave informed consent before participating in this substudy. This study was performed in accordance with the International Conference on Harmonization good clinical practice guidelines and the study protocol was reviewed and approved by the institutional review board or ethics committee at participating sites. The study was approved by the institutional review boards of participating centers and was conducted in accordance with the Declaration of Helsinki.

**Fig. 1:**
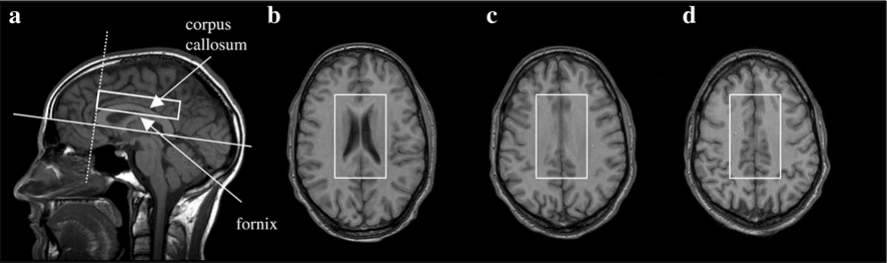
Voxel placement. Voxel positions were **a** a sagittal MRI displaying the single voxel used for MRS angulated parallel to the callosal line, with the bottom edge of the voxel at the intersection of the corpus callosum and the fornix, and the anterior edge of the voxel aligned with the anterior tip of the genu, **b** the most caudal transverse image that intersects the voxel, **c** a slice through the middle of the voxel, and **d** the most superior transverse image intersecting the voxel

Water-suppressed proton spectra were obtained prior to injection of gadolinium (Gd) from a brick-shaped volume of interest centered on the corpus callosum using a 90°–180°–180° (PRESS) sequence (TR 2,000, TE 272, number of acquisitions 128). A water-unsuppressed reference scan to enable correction for eddy currents was obtained immediately after the water-suppressed scan using exactly the same TR, TE, voxel position and shim settings, but using only 16 acquisitions. The voxel measured 50 mm left–right × 70 mm antero-posteriorly × 18 mm superior-inferiorly and was rotated around the left–right axis to be parallel to the sub-callosal line. The voxel was positioned superior-inferiorly so that the inferior margin of the voxel was at the level of the angle of the corpus callosum and the fornix on the mid-sagittal MRI, and antero-posteriorly so that the anterior margin of the voxel was aligned with the anterior tip of the genu (Fig. 1).

In practice, the so-called NAA peak measured by proton MRS in human brain originates from the *N*-acetyl groups (NA) present primarily in NAA, with a small contribution from *N*-acetylaspartylglutamate (NAAG), which also is a neuroaxonal-specific marker. Thus, although the convention is to report the peak as ‘NAA’, the total NAA + NAAG signal is used rather than the signal from NAA alone, because NAA and the much-smaller NAAG resonances are not well resolved at standard field strengths, and the total NAA + NAAG signal is more reliably measured. Results are expressed as the ratio of NAA to total creatine in the VOI.

Quantification of spectra was performed centrally at NeuroRx Research using LCModel [14] and raw digital MRS data that were provided by clinical sites.

## Results

### Baseline characteristics

Baseline characteristics for patients in the main study and the MRS substudy are shown in Table 1. Nineteen patients were randomized to treatment with GA and 15 to placebo. Pretreatment NAA/Cr concentrations in the two groups were the same. Since patients terminated when they relapsed and developed CDMS, the numbers of patients available for follow-up decreased with time over the length of the study. At 12 months, 11 patients treated with GA and nine patients who received placebo remained in the MRS substudy.

**Table 1:** Baseline characteristics of the complete PreCISe cohort, the MRS subcohort, and subjects with 12-month MRS data

Characteristic mean ± SD	PreCISe study		MRS substudy			
			All patients in the substudy		Patients with 12-month MRS data	
	GA 20 mg (*n* = 243)	Placebo (*n* = 238)	GA 20 mg (*n* = 19)	Placebo (*n* = 15)	GA 20 mg (*n* = 11)	Placebo (*n* = 9)
Age (years)	32 ± 6.9	31 ± 7.0	33 ± 7.5	33 ± 7.1	33 ± 9.0	32 ± 8.1
Sex	159 (65 %) ♀	163 (68 %) ♀	14 (74 %) ♀	12 (80 %) ♀	9 (82 %) ♀	9 (100 %) ♀
	84 (35 %) ♂	75 (32 %) ♂	5 (26 %) ♂	3 (20 %) ♂	2 (18 %) ♂	0 ♂
EDSS	1.1 ± 1.0	1.0 ± 1.0	1.3 ± 1.2	1.1 ± 1.1	1.3 ± 1.4	1.2 ± 1.1
NAA/Cr levels			3.3 ± 0.4	3.3 ± 0.6	3.3 ± 0.3	3.3 ± 0.7
# Gd^+^ lesions	1.3 ± 2.8	1.6 ± 2.9	1.9 ± 3	0.9 ± 1	1.7 ± 2.2	0.9 ± 1.2
# T2-weighted lesions	33 ± 34	30 ± 26	37 ± 46	24 ± 23	32 ± 27	29 ± 28
T2 lesion volume (ml)	6.4 ± 7.8	5.7 ± 6.0	7.8 ± 11.1	5.2 ± 4.2	5.8 ± 4.3	6.0 ± 4.7
Brain volume (mm^3^)	1,533 ± 103	1,549 ± 107	1,541 ± 114	1,541 ± 75	1,547 ± 117	1,574 ± 58

### Primary endpoint of the main study

In the overall PreCISe study, patients treated with GA had a 45 % reduction in the risk of developing CDMS compared to patients treated with placebo, and the time to the disease-defining relapse was delayed by approximately 1 year [12].

### Results of the MRS substudy

**Fig. 2:**
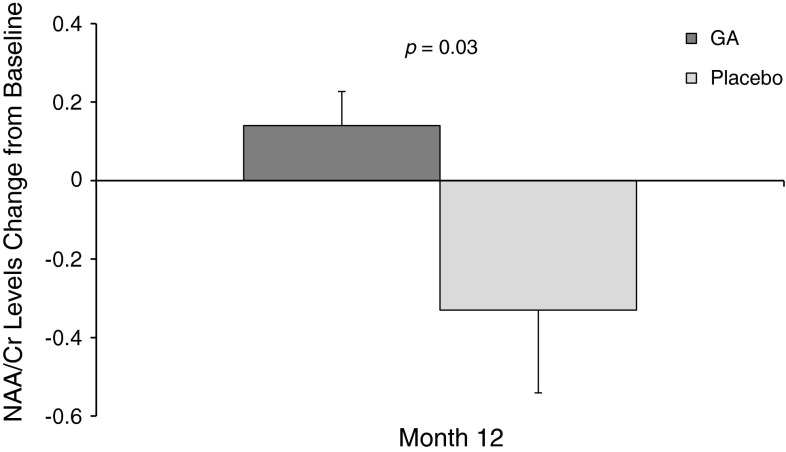
Change from baseline NAA/Cr. Change from baseline in NAA/Cr in patients who had not converted to CDMS and were still in the double-blind phase of the trial at 12 months

In patients treated with GA (*n* = 11), NAA/Cr increased by a mean of 0.14 from baseline to 12 months. In placebo-treated patients (*n* = 9), NAA/Cr decreased by 0.33 from baseline to 12 months (Fig. 2). Changes from baseline NAA/Cr differed significantly between the groups (*p* = 0.03) at month 12.

Analysis of covariance examining the effect of T2-weighted lesions, categorized by the presence or absence of new T2-weighted lesions, showed that increased NAA/Cr was significant in patients who developed new T2-weighted lesions over the course of the study. Scans of these patients were reviewed and it was determined that these new T2-weighted lesions were not located in the VOI for MRS.

For the relatively small number of patients included in this MRS substudy, significant differences between GA treatment and placebo were not found in other MRI outcome measures (including contrast-enhancing lesion frequency, new T2-weighted lesion counts, T2 lesion volumes and percent brain volume change) or in EDSS scores.

## Discussion

In this phase III, randomized, placebo-controlled trial of GA therapy for patients with CIS, patients who received GA showed improvement in brain neuroaxonal integrity relative to patients treated with placebo, who showed the decline expected from natural history studies [6, 7].

The increase in NAA concentrations in patients who were treated with GA is in keeping with a reversal of pre-existing sublethal neuroaxonal injury and associated mitochondrial dysfunction. This phenomenon has been demonstrated experimentally in animals treated with a mitochondrial toxin [15], and has been observed in humans with reversible neuronal injury and dysfunction due to focal inflammatory lesions in MS and mitochondrial encephalopathy associated with lactic acidosis and strokes [5]. Reversible neuronal mitochondrial dysfunction also may underlie the relation between the relative increase in NAA/Cr and new T2 lesion formation. Mitochondrial dysfunction in MS is likely related, at least in part, to an inflammatory environment in the CNS, and patients who are actively forming new T2 lesions are likely to have a more inflammatory environment in their brains. The increased NAA/Cr was not associated with T2 lesion formation within the VOI per se, but rather was related to lesion formation remote from the VOI for spectroscopy. The diffuse nature of this inflammation is an important consideration, as it may be, at least in part, responsible for the widespread neuroaxonal degeneration that occurs in MS.

The absence of a detectable effect of GA on the rate of atrophy in this substudy is not interpretable, as the number of patients was too small to detect a slowing of atrophy. The fact that GA did not just slow the expected decline in NAA, but rather produced an increase in this marker of neuroaxonal integrity provided the statistical power that allowed the effect to be observed by MRS, despite the small number of patients. In addition, acceleration of atrophy after initiation of anti-inflammatory therapy may attenuate the sensitivity to detect a true slowing of atrophy over the first year or two of therapy [16, 17].

This is the first time a treatment effect has been demonstrated in a multicenter, randomized, placebo-controlled clinical trial using the pathologically specific neuroaxonal marker, NAA. Similar findings have been reported previously for RRMS patients treated with GA [9–11], and inconsistently for patients treated with interferon beta [18, 19], but those data are only from small, single-center studies that did not use randomization or placebo controls. Two previous multicenter, randomized, placebo-controlled trials of GA have acquired MRS data in a subset of patients. One study [20] evaluated an oral formulation of GA in patients with relapsing-remitting disease and the other examined subcutaneous administration of GA in patients with primary progressive MS [21]. In both of these trials, the therapy was ineffective on all outcomes, including changes in NAA levels [22]. This is the first study to assess the effect of GA on neuroaxonal integrity in patients with CIS. The difference in findings between the current study, which showed significant improvement in NAA levels in GA-treated patients at 12 months, and the negative outcomes of the GA studies above, may be due to the fact that patients in the current study received GA at an early stage of disease, when subclinical axonal injury is still reversible. Recent observations using magnetic transfer ratio (MTR) as a marker of myelin content suggest that attenuation of demyelination or improvement of remyelination by GA may contribute to axonal preservation [23].

Although absolute and semi-absolute quantification of MRS-measured NAA is often attempted [24], we and others prefer to normalize the NAA signal intensity to that of creatine and phosphocreatine (Cr) in the same voxel. This ratio is straightforward to calculate in the context of multicenter clinical trials and corrects for many sources of variability that can affect estimates of absolute NAA concentration. For example, because Cr is present in virtually all types of brain tissue, but is not present in the cerebrospinal fluid, NAA/Cr ratios are insensitive to the effects of edema, brain atrophy, and sulcal enlargement—pathological changes that have been shown to be present even in patients with CIS suggestive of MS [25, 26]. In addition, changes in the relaxation time of Cr parallel changes in relaxation times of NAA, which at least partially corrects for pathological changes in relaxation times [27]. Correcting for all the above factors in absolute quantification is difficult since it is necessary to determine the volume of tissue from which the MRS signal originates and to correct for changes in T1 and T2 relaxation times of each of the metabolite resonances. Since it is not feasible to measure these quantities in the context of a clinical trial, absolute or semi-absolute quantification would require estimates and assumptions based on healthy subjects or other MS populations. Such assumptions necessarily contain errors that would propagate through the MRS results. The approach of using intravoxel creatine avoids the effect of not knowing these quantities. Although the concentration of Cr may be slightly altered in MS, any alteration is likely to be very small or non-existent in patients with CIS, and are intrinsically accounted for in our analysis, since we report differences in the ratio of NAA/Cr between groups of subjects randomized to either GA or placebo [28].

The observations reported here support neuroprotective activity by GA, and importantly, demonstrate the general feasibility of using MRS to monitor sublethal neuroaxonal injury and its potential response to therapy in the context of multicenter clinical trials.
